# Li_2_(BH_4_)(NH_2_) Nanoconfined in SBA-15 as Solid-State Electrolyte for Lithium Batteries

**DOI:** 10.3390/nano11040946

**Published:** 2021-04-08

**Authors:** Qianyi Yang, Fuqiang Lu, Yulin Liu, Yijie Zhang, Xiujuan Wang, Yuepeng Pang, Shiyou Zheng

**Affiliations:** School of Material Science and Engineering, University of Shanghai for Science and Technology, Shanghai 200093, China; 1826410107@st.usst.edu.cn (Q.Y.); 1826410208@st.usst.edu.cn (F.L.); 193742733@st.usst.edu.cn (Y.L.); 1826410131@st.usst.edu.cn (Y.Z.); 1826410104@st.usst.edu.cn (X.W.); syzheng@usst.edu.cn (S.Z.)

**Keywords:** lithium borohydrides, lithium amides, nanoconfinement, solid electrolytes, all-solid-state batteries

## Abstract

Solid electrolytes with high Li-ion conductivity and electrochemical stability are very important for developing high-performance all-solid-state batteries. In this work, Li_2_(BH_4_)(NH_2_) is nanoconfined in the mesoporous silica molecule sieve (SBA-15) using a melting–infiltration approach. This electrolyte exhibits excellent Li-ion conduction properties, achieving a Li-ion conductivity of 5.0 × 10^−3^ S cm^−1^ at 55 °C, an electrochemical stability window of 0 to 3.2 V and a Li-ion transference number of 0.97. In addition, this electrolyte can enable the stable cycling of Li|Li_2_(BH_4_)(NH_2_)@SBA-15|TiS_2_ cells, which exhibit a reversible specific capacity of 150 mAh g^−1^ with a Coulombic efficiency of 96% after 55 cycles.

## 1. Introduction

Enhancing the energy density and safety of rechargeable lithium batteries is extremely crucial for widespread applications, including electronic vehicles, integrated electronic devices, and smart grids [1,2,3]. Today, the current research hotspot of lithium batteries has been mainly centered on the exploration of advanced electrolytes with high ionic conductivity and electrochemical stability [4,5]. Nevertheless, although traditional organic liquid electrolytes exhibit excellent ionic conductivity, they inevitably suffer from inadequate electrochemical stabilities, dendritic Li growth, and serious safety hazards from battery combustion and explosion [6,7].

In view of these concerns, replacement of liquid electrolytes with mechanically rigid, nonflammable solid-state electrolytes (SSEs) will not only ensure safety during the lithium battery cycling but also significantly suppress dendritic Li formation [8,9,10]. Many families of SSEs with remarkable Li-ion conductivities have been proposed and investigated in the past few decades. Recently developed SSEs, including polymers (PEO-lithium salts [11,12,13]), oxides (perovskite-type [14,15], NASICON [16,17], LISICON [18,19], and garnet-type [20,21]), and thiophosphates (Li_10_GeP_2_S_12_ [22] and Li_2_S-P_2_S_5_ [23,24,25]), show considerably high Li-ion conductivities on the order of 10^−7^ to 10^−3^ S cm^−1^. However, in spite of the high conductivity, most of these SSEs fail to fulfill the requirement of enough electrochemical stability with active electrode materials, thus hampering their further applications in all-solid-state lithium batteries (ASSLBs) [26,27,28].

Lithium borohydrides, members of the family of complex metal hydrides and well-known candidates for hydrogen storage [29,30,31], have drawn intense research interests and proved to be a new class of promising SSEs for ASSLBs because of their excellent Li-ion conduction properties [32,33]. As a representative material, LiBH_4_, in the hexagonal phase (*P6_3_mc*), exhibits high ionic conductivity (10^−3^ S cm^−1^) at 120 °C, while its phase transformation to orthorhombic phase (*Pnma*) occurs below 110 °C, leading to a significant decrease in its Li-ion conductivity (<10^−7^ S cm^−1^ at room temperature (RT)) [34].

Improving the ionic conductivity of LiBH_4_ at low temperature is important for its widespread application in ASSLBs. Matsuo et al. have reported that Li_2_(BH_4_)(NH_2_), formed by the ball milling of LiBH_4_ and LiNH_2_, showed an ionic conductivity four orders of magnitude higher than that of LiBH_4_ at RT, which is attributed to the combination of (BH_4_)^−^ and (NH_2_)^−^ providing more occupation sites for the Li–ion [35]. Similar enhancements in ionic conductivity were realized via phase modifications [21,36,37,38,39]. Additionally, Blanchard et al. demonstrated that nanoconfinement of LiBH_4_ in ordered mesoporous silica scaffolds (MCM-14) notably increases the Li-ion conductivity to 10^−4^ S cm^−1^ at 55 °C owing to the fast anion mobilities of (BH_4_)^−^ near the scaffold wall [40]. Furthermore, it is reported that the ionic conductivity of LiBH_4_–LiNH_2_/metal oxide nanocomposites could be improved by stabilization of a highly conductive phase inside the scaffold pores [41]. However, further in-depth investigation on applying these SSEs in ASSLBs is still highly essential.

In this work, we conducted research on confining highly ion-conductive Li_2_(BH_4_)(NH_2_) in nanopores of the mesoporous silica molecule sieve (SBA-15). The synergic effect generated by the combination of (NH_2_)^−^ substitution and SBA-15 nanoconfinement results in considerably enhanced ionic conductivity and electrochemical stability of Li_2_(BH_4_)(NH_2_)@SBA-15. More importantly, the outstanding Li-ion conduction properties of Li_2_(BH_4_)(NH_2_)@SBA-15 enable the stable cycling of Li||TiS_2_ ASSLBs.

## 2. Materials and Methods

### 2.1. Materials Synthesis

All preparations and manipulations of materials were performed in a glove box with a circulation purifier (Ar atmosphere, <0.1 ppm O_2_ and H_2_O). LiBH_4_ (95%, Sigma-Aldrich, Shanghai, China) and LiNH_2_ (99.95%, Sigma-Aldrich, Shanghai, China) were used as received without further purification. SBA-15 was prepared according to the method introduced in the literature [42] and dried under dynamic vacuum at 250 °C for 12 h prior to use. LiBH_4_ and LiNH_2_ in a molar ratio of 1:1 were ball-milled at 400 rpm for 12 h under an Ar atmosphere. The mixture was then heated at 5 °C min^−1^ to 120 °C and held under a 100-bar H_2_ atmosphere for 12 h to obtain Li_2_(BH_4_)(NH_2_). Li_2_(BH_4_)(NH_2_)@SBA-15 was synthesized by heating a mixture of SBA-15 and milled Li_2_(BH_4_)(NH_2_) with different weight ratios (60:40, 50:50, 30:70, 20:80) under 100-bar H_2_ at 95 °C for 3 h. Afterwards, the sample was cooled and transferred to the Ar-filled glove box.

### 2.2. Structural Characterization

X-ray diffraction (XRD) measurements were conducted with homemade holders on a MiniFlex 600 (Rigaku Corporation, Tokyo, Japan) at a scan rate of 5° min^−1^. Fourier-transform infrared spectroscopy (FTIR) was recorded by a Vector 22 (Bruker Corporation, Billerica, MA, USA) in transmission mode. Scanning electron microscopy (SEM) observations and energy dispersive X-ray spectroscopy (EDS) mapping were performed on a Nova SEM 230 (FEI, Hillsboro, OH, USA) equipped with an X-Max 80 (INCA, Abingdon, UK). The structural information of specific surface areas, pore volumes, and radii were analyzed on an ASAP2020 (Micromeritics Instrument Corporation, Norcross, GA, USA) using the Brunauer–Emmett–Teller (BET) and Barrett–Joyner–Halenda (BJH) methods.

### 2.3. Cell Assemblies and Electrochemical Measurements

All-solid-state cells were assembled using homemade Swagelok-type dies of 10 mm-diameter under 400 MPa pressure. The thickness of the electrolyte pellets was 3 mm for the Li-ion conduction measurement and 0.7 mm for other electrochemical measurements. For the Li||TiS_2_ cells, the anode was Li foil (99.5%) of 6 mm-diameter, and the cathode was a TiS_2_-based composite (8 mg, 50 wt% TiS_2_, 25 wt% Li_2_(BH_4_)(NH_2_)@SBA-15, 25 wt% C) pressed into pellets of 8-mm diameter and less than 0.02 mm thickness.

Electrochemical impedance spectroscopy (EIS), direct current (DC) polarization, and cyclic voltammetry (CV) were conducted on an Interface 1000E (Gamry Instruments, Warminster, England). Galvanostatic charge/discharge (GCD) curves were recorded for the Li||TiS_2_ ASSLBs using a CT2001A battery tester (LAND, Wuhan, China) at different current densities. The conductivity (*σ*) was calculated from the EIS of blocking cells. A depressed semicircle and a linear tail were observed in each Nyquist plot, in which the intersection of the semicircle with the Z’ axis corresponds to the resistance (*R*). The conductivity can be derived from the following equation:

(1) $$\sigma =\frac{d}{AR},$$

where *d* is the thickness, and *A* is the area of the electrolyte pellet. CV measurements of Li||Mo cells were carried out in the voltage range of −0.2–3.5 V with a rate of 0.5 mV s^−1^ at 55 °C, and that of the Li||TiS_2_ cells were measured within 1.6–2.7 V with the same rate and temperature. The electronic transference number was estimated from the ratio of steady current to initial current in the 10 mV potential step curve of a Mo||Mo cell, and the Li-ion transference number was obtained from the same ratio of a Li||Li symmetric cell. The results were calculated from the following equation:

(2) $$t_{e^{-}}=\frac{d\cdot I_{e^{-}}}{A\cdot \Delta V\cdot \sigma_{T}},$$

(3) $$t_{Li^{+}}=\frac{d\cdot I_{Li}+R_{A}}{A\cdot \Delta V\cdot R_{B}\cdot \sigma_{T}},$$

where *d* and *A* are the thickness and area of electrolytes, respectively, $\sigma_{T}$ is the overall conductivity, $I_{e^{-}}$ and $I_{Li^{+}}$ are the steady currents in the DC polarization tests, ΔV is the DC polarization potential, and $R_{B}$ and $R_{A}$ are the resistance before and after polarization, respectively. 

The activation energy (*E_a_*) was calculated from the Nernst–Einstein equation:

(4) $$\ln\left(\sigma_{T}\right)=-\frac{E_{a}}{k_{B}T}+C,$$

where *k_B_* is the Boltzmann constant.

## 3. Results and Discussion

### 3.1. Preparation of Li_2_(BH_4_)(NH_2_)@SBA-15

**Figure 1:**
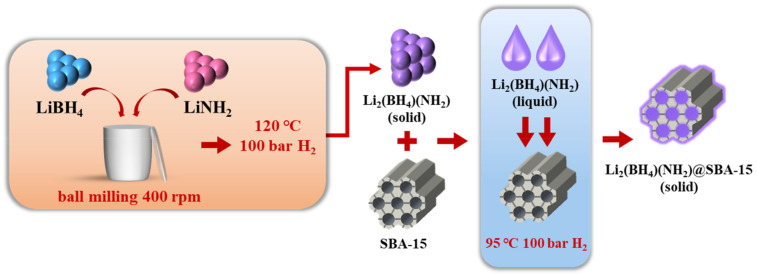
A schematic illustration of the Li_2_(BH_4_)(NH_2_)@SBA-15 synthesis process.

As illustrated in Figure 1, a 3-step preparation process is applied for Li_2_(BH_4_)(NH_2_)@SBA-15 preparing. Firstly, LiBH_4_ and LiNH_2_ with a molar ratio of 1:1 was ball milled to form a mixture. Secondly, the mixture was then heated under 120 °C and 100 bar hydrogen for 12 h to generate Li_2_(BH_4_)(NH_2_). Finally, Li_2_(BH_4_)(NH_2_) is nanoconfined into SBA-15 by heating the mixtures of Li_2_(BH_4_)(NH_2_) and SBA-15 at 95 °C and 100 bar hydrogen for 3 h.

### 3.2. Structures of Li_2_(BH_4_)(NH_2_)@SBA-15

**Figure 2:**
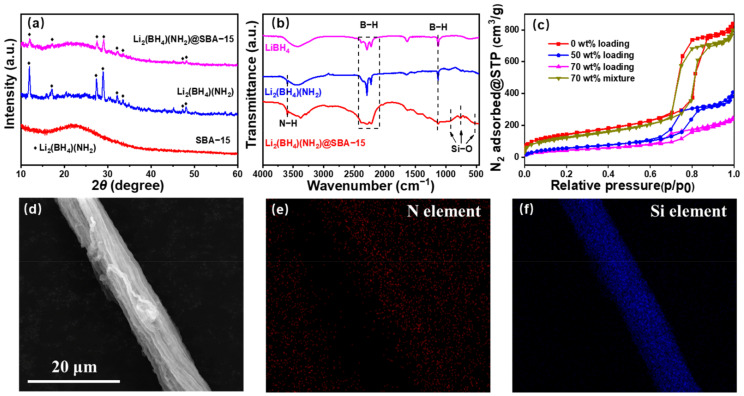
(**a**) X-ray diffraction (XRD) patterns of SBA-15, Li_2_(BH_4_)(NH_2_) and Li_2_(BH_4_)(NH_2_)@SBA-15; (**b**) Fourier-transform infrared (FTIR) spectra of Li_2_(BH_4_)(NH_2_)@SBA-15, Li_2_(BH_4_)(NH_2_) and LiBH_4_; (**c**) N_2_ absorption isotherms of SBA-15, Li_2_(BH_4_)(NH_2_)/SBA-15, and Li_2_(BH_4_)(NH_2_)@SBA-15; (**d**) scanning electron microscopy (SEM) images of Li_2_(BH_4_)(NH_2_)@SBA-15; (**e**,**f**) energy dispersive X-ray spectroscopy (EDS) images of Li_2_(BH_4_)(NH_2_)@SBA-15 illustrating the distribution of characteristic elements N and Si, respectively.

XRD was employed to clarify the phase composition of Li_2_(BH_4_)(NH_2_)@SBA-15. As demonstrated in Figure 2a, the main peaks appearing in the Li_2_(BH_4_)(NH_2_) curve can be well-indexed to the pure phase at RT, suggesting a successful preparation. The characteristic peaks of Li_2_(BH_4_)(NH_2_) clearly remain in Li_2_(BH_4_)(NH_2_)@SBA. These XRD patterns confirm that no phase transformation occurred in Li_2_(BH_4_)(NH_2_) after loading it into SBA-15.

Considering that amorphous phases, which cannot be detected by XRD, may be generated from the decomposition of Li_2_(BH_4_)(NH_2_) [43], FTIR was used to further identify the phase composition of Li_2_(BH_4_)(NH_2_)@SBA-15. As can be seen in the FTIR spectra (Figure 2b), all peaks appearing in Li_2_(BH_4_)(NH_2_) belong to the stretching vibration of B–H and N–H. After loading Li_2_(BH_4_)(NH_2_) into SBA-15, the newly appearing peaks in Li_2_(BH_4_)(NH_2_)@SBA-15 can be assigned to the Si–O bond, and no signal for decomposition byproducts of Li_2_(BH_4_)(NH_2_), such as Li–B–N compounds or B, is detected. These results are well in accordance with the previous discussion from the XRD patterns and indicate that the decomposition of Li_2_(BH_4_)(NH_2_) is totally suppressed by the presence of high-pressure hydrogen during the materials synthesis.

To further confirm the effective confinement of Li_2_(BH_4_)(NH_2_) into the SBA-15 mesopores by the melting–infiltration method, BET is applied to characterize the porosity parameters, as infiltration of Li_2_(BH_4_)(NH_2_) into SBA-15 may change the porosity of SBA-15. The porosities of SBA-15, 40 wt% Li_2_(BH_4_)(NH_2_)@SBA-15, 70 wt% Li_2_(BH_4_)(NH_2_)@SBA-15 and a 70 wt% Li_2_(BH_4_)(NH_2_)/SBA-15 mixture were measured via nitrogen physisorption (normalized to 1g of SBA-15). Interpreted from the results in Figure 2c, the typical steep capillary condensation step and a hysteresis loop are observed in the pure SBA-15, agreeing well with the results for SBA-15 elsewhere [44]. Marked reductions in nitrogen absorption amounts were detected for the two melt-infiltrated Li_2_(BH_4_)(NH_2_)@SBA-15 samples, while that of the Li_2_(BH_4_)(NH_2_)/SBA-15 mixture generally remained unchanged. Combining these results with the pore parameter of SBA-15, we can conclude that the melted Li_2_(BH_4_)(NH_2_) was successfully infiltrated into the mesopores of SBA-15 via a capillary action, and 90% of the SBA-15 mesopores were filled with 70 wt%Li_2_(BH_4_)(NH_2_) (Table S1).

SEM and EDS mapping were employed for in-depth investigations of the morphology and elemental distribution of Li_2_(BH_4_)(NH_2_)@SBA-15 after the melting–infiltration process. In Figure 2d, the typical morphology of the SBA-15 scaffolds is observed, indicating that it remained almost intact after being infiltrated (Figure 2d). The EDS mapping images (Figure 2e,f) show that the distribution patterns of characteristic elements (N and Si, respectively) match well with the shapes of SBA-15 fibers, suggesting that Li_2_(BH_4_)(NH_2_) was evenly distributed into the mesopores of the SBA-15 scaffolds.

The above results demonstrate that the pure Li_2_(BH_4_)(NH_2_) phase was formed by partially substituting (BH_4_)^−^ with (NH_2_)^−^ at 120 °C. The possible decomposition of Li_2_(BH_4_)(NH_2_) during the synthesis process was suppressed by the high-pressure hydrogen atmosphere. Furthermore, the melting–infiltration method at 95 °C successfully enables this new phase to be homogeneously nanoconfined into the mesoporous SBA-15.

### 3.3. Electrochemical Performances

**Figure 3:**
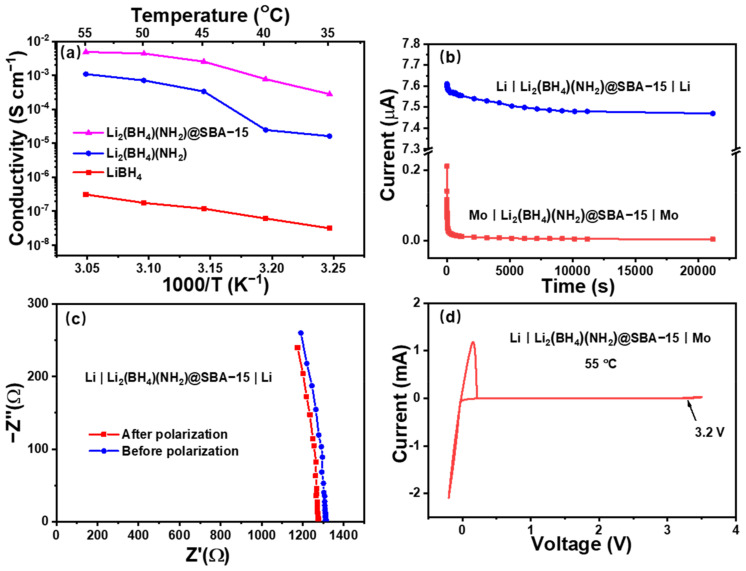
(**a**) Temperature-dependent conductivities of LiBH_4_, Li_2_(BH_4_)(NH_2_)and Li_2_(BH_4_)(NH_2_)@SBA-15; (**b**,**c**) electron and Li-ion transference numbers of Li_2_(BH_4_)(NH_2_)@SBA-15, respectively; (**d**) cyclic voltammetry (CV) measurements of Li_2_(BH_4_)(NH_2_)@SBA-15.

The ionic conductivities of LiBH_4_, Li_2_(BH_4_)(NH_2_), and Li_2_(BH_4_)(NH_2_)@SBA-15 with different loading contents (40 wt%, 50 wt%, 70 wt%, and 80 wt%) were determined from the temperature-dependent EIS (Figure S1). The temperature range is set to be 35 to 55 °C, due to the thermostability of the electrolytes (Figure S2). As displayed in Figure 3a, Li_2_(BH_4_)(NH_2_) exhibits a remarkably enhanced conductivity of 1.6 × 10^−5^ S cm^−1^ at 35 °C, which is generally three orders of magnitude higher than that of the host hydride LiBH_4_. This value even reaches 1 × 10^−3^ S cm^−1^ at 55 °C. As for Li_2_(BH_4_)(NH_2_)@SBA-15, its conductivities in the same temperature range (from 35 °C to 55 °C) are further improved by the nanoconfinement, from 2.8 × 10^−4^ S cm^−1^ to 5.0 × 10^−3^ S cm^−1^. These increased conductivities can be attributed to the synergetic effect of (NH_2_)^−^ substitution and SBA-15 nanoconfinement. Moreover, the conductivity of Li_2_(BH_4_)(NH_2_)@SBA-15 varies with the different loading contents of Li_2_(BH_4_)(NH_2_), among which the maximum effective loading content (70 wt%) generates the most distinguished enhancement in conductivity (Figure S3). The *E_a_* of Li_2_(BH_4_)(NH_2_)@SBA-15 is calculated to be 0.49 eV, which is typical for superior ionic conductors (<0.5 eV) [45].

The transference numbers of Li_2_(BH_4_)(NH_2_)@SBA-15 can be further characterized by DC polarization and EIS. Figure 3c presents the results of DC polarization tested on a Mo|Li_2_(BH_4_)(NH_2_)@SBA-15|Mo cell at 55 °C, showing that the steady current at 10 mV was extremely small, which corresponds to an electronic transference number very close to 0. Additionally, the Li-ion transference number of Li_2_(BH_4_)(NH_2_)@SBA-15 was calculated to be 0.97, from the initial and steady currents, as well as the resistances recorded before and after polarization of Li|Li_2_(BH_4_)(NH_2_)@SBA-15|Li cells (Figure 3d). These results combined with the aforementioned ionic conductivities suggest that Li_2_(BH_4_)(NH_2_)@SBA-15 possesses appreciable Li-ion conducting properties.

These results combined with the XRD patterns and FTIR spectra indicate the enhanced conduction properties can be well attributed to the successful incorporation of Li_2_(BH_4_)(NH_2_) into the mesopores of SBA-15. First, Li_2_(BH_4_)(NH_2_) itself has high Li-ion conductivity, which provides more occupation sites for mobile Li-ions than LiBH_4_. Second, the SBA-15 nanoconfinement generate large Li_2_(BH_4_)(NH_2_)/SiO_2_ interface, which may result in a highly Li-ion conductive region due to the interface effect, as illustrated in literatures published elsewhere [46,47].

High ionic conductivity is one favorable requirement for an SSE. Besides this, the electrochemical stability and electrode compatibility of Li_2_(BH_4_)(NH_2_)@SBA-15 also play essential roles in its application to ASSLBs. To test the electrochemical stability of Li_2_(BH_4_)(NH_2_)@SBA-15, CV measurements were performed on Li|Li_2_(BH_4_)(NH_2_)@SBA-15|Mo cells at 55 °C (Figure 3b). In the −0.2 V to 3.5 V curve, redox peaks are only observed near 0 V and 3.2 V, which are related to the Li plating/stripping reaction on the Mo electrode and decomposition of Li_2_(BH_4_)(NH_2_), respectively. This result indicates that the apparent electrochemical stability window of Li_2_(BH_4_)(NH_2_)@SBA is 0 to 3.2 V.

**Figure 4:**
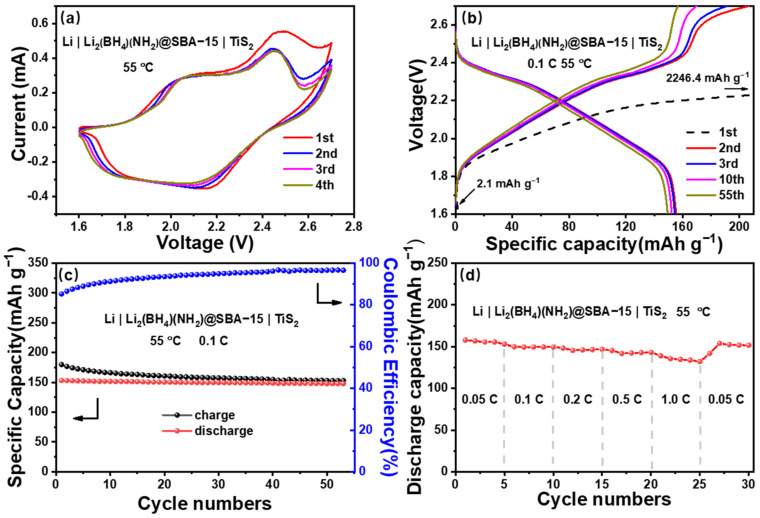
The electrochemical stabilities and cycling performances of Li||TiS_2_: (**a**) CV measurements within the potential range of 1.6–2.7 V; (**b**) charge/discharge profiles in the 1st, 2nd, 3rd, 10th, and 55th cycles; (**c**) cycling performances at 0.1 C and 55 °C; (**d**) rate capability behavior for 0.05 C–1 C.

Considering the electrochemical window of Li_2_(BH_4_)(NH_2_)@SBA-15 and the electrochemical compatibility of TiS_2_ with the LiBH_4_-based electrolytes [39,48], we employed Li||TiS_2_ cells to evaluate the compatibilities of Li_2_(BH_4_)(NH_2_)@SBA-15 with the electrode materials. In the CV measurements (Figure 4a) at 55 °C, the two adjacent peaks appearing at 2.2 and 1.8 V in anodic sweep correspond to the lithiation which can be described by the reaction TiS_2_ + Li^+^ + e^−^ → LiTiS_2_. Moreover, three oxidative peaks are observed in the cathodic sweep at 2.0, 2.4 and 2.7 V. The first two oxidative peaks can be interpreted by the delithiation reaction of LiTiS_2_ (LiTiS_2_ → TiS_2_ + Li^+^ + e^−^), while the third one appearing at 2.7 V is due to the irreversible side-reaction which formed a solid electrolyte interface (SEI) film on the cathode side. In the subsequent cathodic sweeps, the peak intensities at 2.7 V decrease obviously, while integration areas and other peak positions remain almost unchanged, indicating that the side reaction was prevented by the SEI and the Li-ion insertion, and the extraction was relatively stable during cycling. Overall, the CV curves reflect that Li_2_(BH_4_)(NH_2_)@SBA-15 is highly compatible with the Li and TiS_2_ electrodes.

The typical charge/discharge profiles and cycling performances of Li||TiS_2_ were profiled by GCD measurements at 0.1 C and 55 °C (Figure 4b,c). From the figures, the initial discharge capacity is relatively low, owing to the self-discharge reaction between TiS_2_ and Li_2_(BH_4_)(NH_2_)@SBA-15. The subsequent initial charging capacity is far exceeding the theoretical capacity of TiS_2_ (239 mAh g^−1^), which is similar to previous researches involving the same Li and TiS_2_ electrode [49]. This phenomenon is attributed to the irreversible oxidative decomposition of Li_2_(BH_4_)(NH_2_)@SBA-15 during the charging process.

Two plateaus at 2.3 and 1.9 V are present in the discharging curves in the overall cycling operation, and the discharging capacity is well retained at 150 mAh g^−1^ during the subsequent 55 cycles. In the second charging curve, three voltage plateaus are observed at 1.9, 2.3 and 2.6 V, with the total specific capacities of 207 mAh g^−1^. Subsequently, the third voltage plateau gradually diminishes thereafter, which is in accordance with the CV curves. A stable charging capacity at 155 mAh g^−1^ was produced during the following 55 cycles. The Coulombic efficiency of the Li||TiS_2_ cell gradually increased after the formation of a stable SEI and was maintained at 96%.

Displayed in Figure 4d is the rate capability behavior of the Li||TiS_2_ cells, determined by increasing the cycling charge/discharge rates every 5 cycles from 0.05 C to 1 C and then decreasing the rate to 0.05 C. The reversible discharge capacities measured at 0.05 C, 0.1 C, 0.2 C, 0.5 C, and 1 C are 155, 149, 146, 142, and 135 mAh g^−1^, respectively. Significant decreases in capacities delivered at increasingly higher current densities are not observed. In addition, the capacity of the Li||TiS_2_ cell was able to recover to 152 mAh g^−1^ at 0.05 C after cycling at high rates. These properties suggest that the (NH_2_)^−^ substitution and SBA-15 nanoconfinement are able to synergically improve the ionic conductivity of Li_2_(BH_4_)(NH_2_)@SBA-15 as a promising SSE for ASSLBs.

## 4. Conclusions

In summary, we have demonstrated the successful preparation of Li_2_(BH_4_)(NH_2_)@SBA-15 by (NH_2_)^−^ substitution and SBA-15 nanoconfinement (a comparison with relevant materials is show in Table 1). Li_2_(BH_4_)(NH_2_)@SBA-15 with 70 wt% loading content exhibited the most significantly enhanced electric conductivity of 2.8 × 10^−4^ S cm^−1^ to 5.0 × 10^−3^ S cm^−1^ in the temperature range of 35–55 °C. The synergic effect of (NH_2_)^−^ substitution and SBA-15 nanoconfinement also provided an extremely low electronic transference number and a Li-ionic transference number of 0.97 at 55 °C, as well as an apparent electrochemical window of 0 to 3.2 V. Furthermore, overall electrochemical performances of Li_2_(BH_4_)(NH_2_)@SBA-15 were investigated by application in Li||TiS_2_ ASSLBs. The Li||TiS_2_ cell delivered a reversible specific capacity of 150 mAh g^−1^ with a Coulombic efficiency of 96% after 55 cycles. The capacities were 155, 149, 146, 142, and 135 mAh g^−1^ at 0.05 C, 0.1 C, 0.2 C, 0.5 C, and 1 C rates, respectively. Moreover, the appearance of an additional plateau in the initial charging processes indicated a side reaction between Li_2_(BH_4_)(NH_2_)@SBA-15 and cathode to form a protective SEI. Further investigations on the identification of this SEI will be carried out in our future works.

## Figures and Tables

**Table 1:** Li-ion conduction properties of present LiBH_4_-based materials and their present applications in all-solid-state lithium batteries (ASSLBs).

LiBH_4_-Based Materials	$\sigma_{55{}^{\circ}C}{\;}^{1}\;(S\;\mathrm{cm}{}^{-1})$	*E_a_*^2^ (eV)	Electrochemical Window (V vs. Li/Li^+^)	Applications in ASSLBs	Ref.
LiBH_4_ (*P6_3_mc*)	10^−3^ (120 °C)	0.53	5	Li||TiS_2_	[34,48]
LiBH_4_ (*Pnma*)	10^−8^	0.69	5	-	[34]
LiBH_4_@SBA-15	9 × 10^−4^	0.43	3.5	Li||S	[40,50]
Li_2_(BH_4_)(NH_2_)	9 × 10^−4^	0.66	-	-	[32,35]
Li(NH_3_)*_n_*BH_4_ (0.5 ≤ *n* ≤ 1)	10^−3^–10^−^^2^ (40 °C)	-	4	-	[37]
LiBH_4_–LiX (X = Cl, Br and I)	10^−6^−10^−4^	0.39–0.64	-	LiNbO_3_-coated LiCoO_2_/KB/80Li_2_S 20P_2_S_5_||Li	[32,49]
LiBH_4_−MgO composites	9 × 10^−3^	0.29	2.2	Li||TiS_2_	[39]
LiBH_4_–LiNH_2_/metal oxide nanocomposites	10^−3^	0.86–0.90	-	-	[41]
Li_4_(BH_4_)_3_I@SBA-15	8 × 10^−3^	0.46	5	Li||Li_4_Ti_5_O_12_, Li||S, Li||LiCoO_2_	[44]
Li_2_(BH_4_)(NH_2_)@SBA-15	5 × 10^−3^	0.49	3.2	Li||TiS_2_	-

^1^ Li-ion conductivity at 55 °C; ^2^ Activation energy for Li-ion conduction.

## Data Availability

The data presented in this study are available on the request from the corresponding writer.

## Supplementary Materials

The following are available online at https://www.mdpi.com/article/10.3390/nano11040946/s1. Figure S1: A representative Nyquist plot of Li_2_(BH_4_)(NH_2_)@SBA-15 derived from the electrochemical impedance spectroscopy (EIS) tests with an equivalent circuit, Figure S2. Thermogravimetric (TG) curve of Li_2_(BH_4_)(NH_2_)@SBA-15, Figure S3: Conductivities of Li_2_(BH_4_)(NH_2_)@SBA-15 with different loading contents of 40 wt%, 50 wt%, 70 wt% and 80 wt% at various temperatures, Table S1: Pore parameters of SBA-15, Li_2_(BH_4_)(NH_2_)@SBA-15 samples and Li_2_(BH_4_)(NH_2_)/SBA-15 mixtures.
