# Supplementary material for: Li2(BH4)(NH2) Nanoconfined in SBA-15 as Solid-State Electrolyte for Lithium Batteries
Source: Nanomaterials (Basel). 2021 Apr 8;11(4):946. doi: 10.3390/nano11040946 (PMC8068180; doi:10.3390/nano11040946)
Supplement: Supplementary file 1 [file nanomaterials-11-00946-s001.pdf]

nanomaterials

Supplementary Materials

$Li_2(BH_4)(NH_2)$ Nanoconfined in SBA-15 as Solid-State Electrolyte for Lithium Batteries

Qianyi Yang, Fuqiang Lu, Yulin Liu, Yijie Zhang, Xiujuan Wang, Yuepeng Pang * and Shiyou Zheng

School of Material Science and Engineering, University of Shanghai for Science and Technology, Shanghai 200093, China; 1826410107@st.usst.edu.cn (Q.Y.); 1826410208@st.usst.edu.cn (F.L.); 193742733@st.usst.edu.cn (Y.L.); 1826410131@st.usst.edu.cn (Y.Z.); 1826410104@st.usst.edu.cn (X.W.); syzheng@usst.edu.cn (S.Z.)

* Correspondence: pangyp@usst.edu.cn

The Nyquist plot of $Li_2(BH_4)(NH_2)$@SBA-15 can be well fitted using the equivalent circuit model shown in the inset. In the equivalent circuit, R represents the resistance of ionic transformation and W represents Warburg resistance generated by Li-ion diffusion. They are in series connection and then in parallel connection with a constant phase element (CPE).

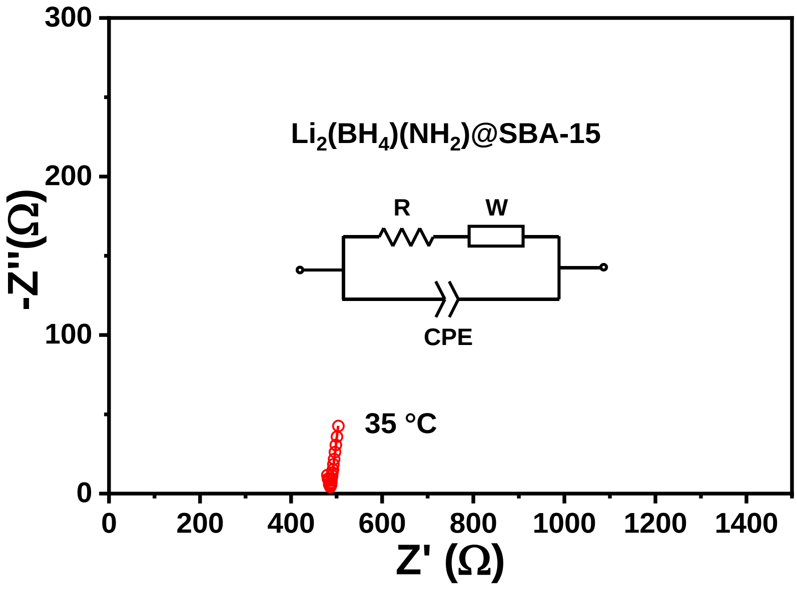

Figure 1. A representative Nyquist plot of $Li_2(BH_4)(NH_2)$@SBA-15 derived from the electrochemical impedance spectroscopy (EIS) tests with an equivalent circuit.

The thermogravimetric (TG) curve of $Li_2(BH_4)(NH_2)$@SBA-15 presents a marked weight loss occurred near 75 °C, and then a 9 wt% weight loss is observed in the temperature range of 75 to 500 °C, corresponding a hydrogen desorption at these high temperatures.

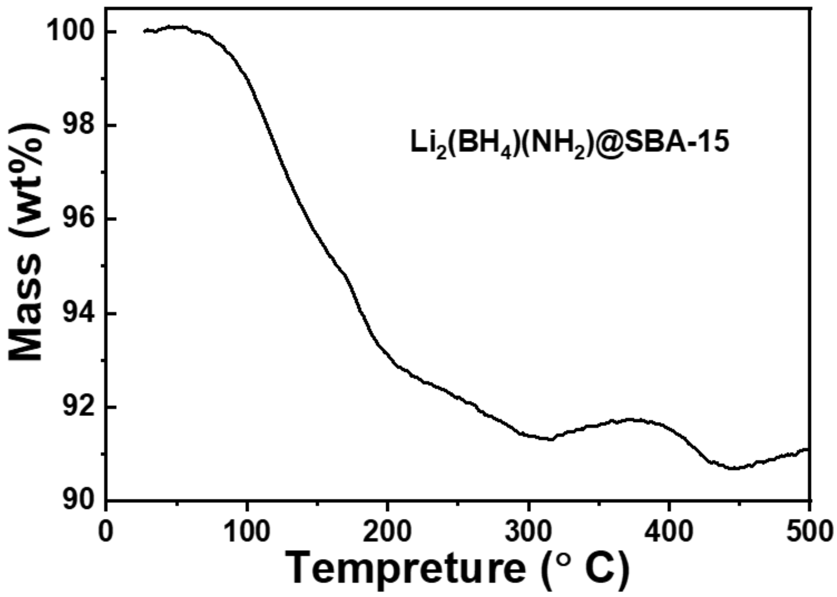

Figure 2. Thermogravimetric (TG) curve of $Li_2(BH_4)(NH_2)$@SBA-15.

Temperature-dependent conductivities of $Li_2(BH_4)(NH_2)$@SBA-15 with different loading contents show the 70 wt% loading content improves the conductivities most significantly, because lower loading contents renders isolating empty mesopores of SBA-15 to a large extent, and higher loading contents leads to mesopores of SBA-15 blocked by $Li_2(BH_4)(NH_2)$.

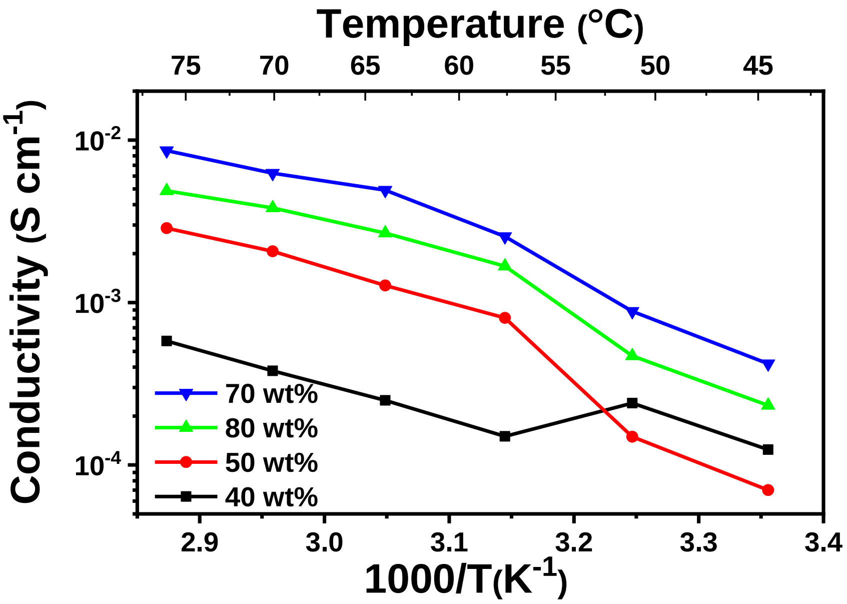

Figure 3. Conductivities of $Li_2(BH_4)(NH_2)$@SBA-15 with different loading contents of 40 wt%, 50 wt%, 70 wt% and 80 wt% at various temperature.

The pore parameters show that both surface area and pore volume decrease considerably with increasing loading contents of $Li_2(BH_4)(NH_2)$. For instance, a 90% reduction in the Brunauer-Emmett-Teller (BET) surface area and Barrett-Joyner-Halenda (BHJ) pore volume are found in the 70 wt% loading sample, while the pore parameters do not change in the mixture sample.

Table 1. Pore parameters of SBA-15, $Li_2(BH_4)(NH_2)$@SBA-15 samples and $Li_2(BH_4)(NH_2)$/SBA-15 mixtures.

**sample**	**BET surface area (m^2 g^{-1})**	**BJH pore volume (cm^3 g^{-1})**
SBA-15	541	1.13
$Li_2(BH_4)(NH_2)$@SBA-15 40 wt% loading	300	0.52
$Li_2(BH_4)(NH_2)$@SBA-15 70 wt% loading	54	0.11
$Li_2(BH_4)(NH_2)$@SBA-15 70 wt% mixture	508	1.09
